# Augmented Reality, Surgical Navigation, and 3D Printing for Transcanal Endoscopic Approach to the Petrous Apex

**DOI:** 10.1177/2473974X18804492

**Published:** 2018-10-29

**Authors:** Samuel R. Barber, Kevin Wong, Vivek Kanumuri, Ruwan Kiringoda, Judith Kempfle, Aaron K. Remenschneider, Elliott D. Kozin, Daniel J. Lee

**Affiliations:** 1 Department of Otolaryngology–Head and Neck Surgery, University of Arizona College of Medicine, Tucson, Arizona, USA; 2 Eaton Peabody Laboratories, Massachusetts Eye and Ear Infirmary, Boston, Massachusetts, USA; 3 Department of Otolaryngology–Head and Neck Surgery, Massachusetts Eye and Ear Infirmary, Boston, Massachusetts, USA; 4 Department of Otology and Laryngology, Harvard Medical School, Cambridge, Massachusetts, USA

**Keywords:** 3D printing, simulation, navigation, skull base, transcanal, endoscopic, residency education, augmented reality

## Abstract

Otolaryngologists increasingly use patient-specific 3-dimensional (3D)–printed anatomic physical models for preoperative planning. However, few reports describe concomitant use with virtual models. Herein, we aim to (1) use a 3D-printed patient-specific physical model with lateral skull base navigation for preoperative planning, (2) review anatomy virtually via augmented reality (AR), and (3) compare physical and virtual models to intraoperative findings in a challenging case of a symptomatic petrous apex cyst. Computed tomography (CT) imaging was manually segmented to generate 3D models. AR facilitated virtual surgical planning. Navigation was then coupled to 3D-printed anatomy to simulate surgery using an endoscopic approach. Intraoperative findings were comparable to simulation. Virtual and physical models adequately addressed details of endoscopic surgery, including avoidance of critical structures. Complex lateral skull base cases may be optimized by surgical planning via 3D-printed simulation with navigation. Future studies will address whether simulation can improve patient outcomes.

Three-dimensional (3D) printing is a useful tool for surgical planning.^1^Surgeons use patient-specific 3D-printed physical models for preoperative planning,^2,3^such as custom plates or surgical guides.^4,5^Recent 3D-printed simulators render complex anatomy accurately.^1,5-8^Augmented reality (AR) projects virtual objects into real-life environments. Although AR applications originated over a decade ago,^9,10^feasibility was limited by ergonomics and accurate patient/image registration.^11^Current technology facilitates conversion of medical imaging to 3D models in AR for mobile devices.^9^

Combining AR with surgical navigation enables personalized preoperative planning. In otologic surgery, virtual cues could identify structures, such as vessels and cranial nerves along the skull base. To date, isolated reports describe personalized preoperative planning.^12,13^Furthermore, no reports discuss planning via virtual and physical surgical simulations with concurrent navigation for a complete visuospatial and tactile experience. Herein, we describe a challenging case of a petrous apex cyst accessible through a subcochlear surgical corridor. Virtual rendering, 3D printing, and navigation enabled a safe transcanal endoscopic approach.

## Methods

### Imaging

**Figure 1.**
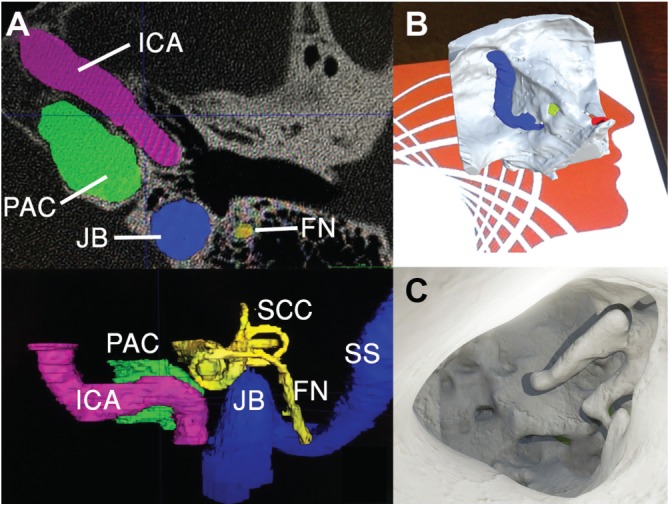
Left ear 3-dimensional (3D) reconstruction. (A) Manual segmentation from computed tomography images into 3D meshes using ITK-SNAP. (B, C) Augmented reality mobile phone application visualized anatomy preoperatively, registered with target image. FN, facial nerve; ICA, internal carotid artery; JB, jugular bulb; PAC, petrous apex cyst; SCC, semicircular canal; SS, sigmoid sinus.

A 48-year-old pilot presenting with left-sided otalgia, tinnitus, and vertex headache exacerbated by elevation changes mid-flight was found to have an ipsilateral petrous apex cyst. No cranial nerve deficits were present. Computed tomography (CT) images were obtained on a SOMATOM Definition AS (Siemens, Munich, Germany) with 0.6-mm slice thickness, 120 kVp, and 1000-ms exposure time. Imaging revealed a cystic mass in the left petrous apex measuring 1.8 × 1.1 × 1.2cm with medial and anterior bony dehiscence abutting the horizontal portion of the carotid artery (**Figure 1A**). The study was deemed exempt by the Massachusetts Eye and Ear Institutional Review Board.

### 3D Modeling

Manual segmentation, the process by which the digital image was partitioned into individual components to isolate anatomic structures, was performed to create 3D models (meshes) from highlighted voxels in DICOM images in ITK-SNAP.^14^Bone, vessel, and nerve were segmented into separate meshes, exported as stereolithography (STL) files, and postprocessed with smoothing filters.

### Augmented Reality

Meshes were imported into Unity v5.6 (San Francisco, California) with Vuforia (Needham, Massachusetts). Custom-scripted user controls enabled zoom, rotation, and opacity changes to surgically oriented models.

### 3D Printing and Navigation Preparation

**Figure 2.**
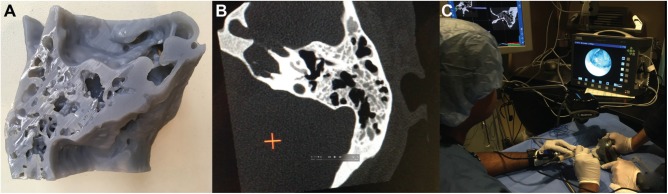
(A) A 3-dimensional (3D) print of temporal bone used for preoperative simulation. (B) Computed tomography scan of a 3D print was a 1:1 match with the original and able to be registered for navigation. (C) Transcanal approach to petrous apex was simulated on 3D print with navigation.

The temporal bone STL file was 3D-printed directly using a Form2 with 25-µm resolution (FormLabs, Somerville, Massachusetts) (**Figure 2A**). Six titanium screws were instrumented as fiducial markers, and another axial CT scan was performed.

### Navigation

Original and 3D-printed CT scans were imported onto a Stealth3D workstation (Medtronic, Minneapolis, Minnesota). Image registration compared structural differences between patient and 3D-printed anatomy. Calibration used fiducial markers. For visualization, rigid otoendoscopes were coupled to high-definition video (Karl Storz, Tuttlingen, Germany).

### Preoperative Simulation

The navigation workstation and 3D-printed model were brought to the surgical training laboratory with a full operating theater setup. The 3D-printed model provided a tactile representation of surgical anatomic landmarks. Following registration, navigation used the original CT data. The procedure was performed as would be done in surgery.

## Results

### 3D Modeling and Augmented Reality

Anatomy was segmented with structures comprising temporal bone, petrous apex cyst, internal carotid artery, jugular bulb, sigmoid sinus, cranial nerves VII/VIII, cochlea, and vestibular apparatus (**Figure 1A**). Physically based rendering materials were applied, and an AR app built for Android OS allowed the interrelationship of critical landmarks to be evaluated from a virtual transcanal view (**Figure 1B**,**C**).

### Preoperative Simulation

Surgical simulation using navigation was performed on the 3D-printed model preoperatively (**Figure 2**). During fiducial registration, the 3D-printed model was a 1:1 match with original CT imaging, with a 0.7-mm margin of error in Stealth3D (**Figure 2B**). Petrous apex cyst access and drainage was performed via a transcanal, endoscopic-assisted (3-mm diameter, 14-cm rigid endoscope, 0, 30-degree) infracochlear approach coupled with navigation (**Figure 2B**,**C**). A limited inferior canalplasty was performed for hypotympanum access. Navigation remained accurate without physical model landmarks deviating from observed coordinates. Of note, during simulation, the carotid canal was encountered anteriorly by the 1-mm burr. This was confirmed using navigation probes placed at the defect.

### Live Surgery

**Figure 3.**
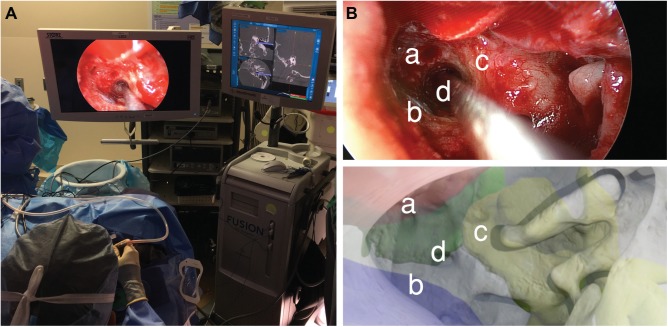
(A) Intraoperative photo of the live surgery performed using a transcanal endoscopic approach. (B) Comparison of intraoperative (upper panel) with virtual, preoperative otoendoscopic views (lower panel) demonstrated that the virtual render predicted the trajectory of the real surgical approach based on structures at risk: (a) internal carotid artery, (b) jugular bulb, (c) basal turn of the cochlea, and (d) access to petrous apex cyst.

Anatomic constraints observed during simulation provided insight for live surgery. An optimized transcanal endoscopic approach achieved successful cyst drainage while avoiding the carotid artery anteriorly, jugular bulb inferiorly, and basal turn of the cochlea superiorly within a 1.5-mm surgical corridor (**Figure 3B**). There were no perioperative complications and the patient experienced symptomatic relief 1 year postoperatively.

## Discussion

A personalized 3D-printed temporal bone was successfully fabricated and coupled with surgical navigation. The 3D-printed model was a high-fidelity replica of patient-specific anatomy, evidenced by registration with original CT imaging and comparisons between simulation and intraoperative findings. Although data loss can occur during segmentation, postprocessing, and 3D printing, the margin of error along landmarks was 0.7 mm, close to 0.6-mm cuts. For delicate lateral skull base microanatomy, results exceeded expectations. Moreover, small open structures, including mastoid air cells and foramina, remained patent.

That the carotid canal was encountered during simulation underscores the value of preoperative surgical planning. Subjectively, 3D-printed bone had good haptic feedback during drilling, and simulation strongly aided in preparation. However, further cases are required to demonstrate improved safety using preoperative planning.

Several studies investigated preoperative planning in otology; however, no studies used multiple formats, including AR.^2,12,13,15^Spine surgery studies involving cadaveric and 3D-printed models demonstrated decreased error with instrumentation.^16,17^Within otolaryngology, surgical planning using combined physical/virtual models may ultimately optimize patient outcomes.

## Conclusion

Complex lateral skull base cases may be optimized by surgical planning using AR and 3D-printed simulation with concurrent navigation. High-fidelity patient-specific models are fabricated using consumer technology. Future studies will address whether simulation can improve outcomes, including patient safety.

## Author Contributions

**Samuel R. Barber**, study design and conduct, collection, analysis, interpretation, and writing; **Kevin Wong**, study design and conduct, collection, analysis, interpretation, and writing; **Vivek Kanumuri**, study design and conduct, collection, analysis, interpretation, and writing; **Ruwan Kiringoda**, study design and conduct, collection, analysis, interpretation, and writing; **Judith Kempfle**, study design and conduct, collection, analysis, interpretation, and writing; **Aaron K. Remenschneider**, study design and conduct, collection, analysis, interpretation, and writing; **Elliott D. Kozin**, study design and conduct, collection, analysis, interpretation, and writing; **Daniel J. Lee**, study design and conduct, collection, analysis, interpretation, and writing.

## Disclosures

**Competing interests:** None.

**Sponsorships:** None.

**Funding source:** None.
